# Clara cell 16 KDa protein mitigates house dust mite-induced airway inflammation and damage via regulating airway epithelial cell apoptosis in a manner dependent on HMGB1-mediated signaling inhibition

**DOI:** 10.1186/s10020-021-00277-4

**Published:** 2021-02-04

**Authors:** Meixuan Liu, Jingjing Lu, Qian Zhang, Yunxuan Zhang, Zhongliang Guo

**Affiliations:** 1 Shanghai East Clinical Medical College, Nanjing Medical University, Shanghai, 200123 China; 2 Department of Respiratory Medicine, Shanghai East Hospital, Tongji University School of Medicine, Shanghai, 200123 China; 3 Department of Pharmacy, Huadong Hospital, Fudan University, Shanghai, 200040 China

**Keywords:** CC16, HMGB1, House dust mite-induced asthma, Airway epithelial cell, Apoptosis

## Abstract

**Background:**

House dust mite (HDM) inhalation can cause airway epithelial damage which is implicated in the process of airway inflammation in asthma. High mobility group box 1 (HMGB1) is critically required for cellular damage and apoptosis as an important endogenous danger signal. Recently, Clara cell 16KDa protein (CC16) has been identified to exert anti-inflammatory and immunomodulatory influence in various injury-related diseases model. However, little is known about its ability to protect against airway epithelial injury in allergic asthma. This study was aimed to clarify the protective roles of CC16 on airway epithelia in HDM-induced asthma and the regulation of HMGB1 by CC16.

**Methods:**

Mice were sensitized and challenged by HDM extract and administrated intranasally with CC16 (5 μg/g or 10 μg/g) or saline in the challenged period. The BEAS-2B human airway epithelial cell line were cultured with CC16 or the control vehicle and then exposed to HDM. Knockdown or overexpression of HMGB1 was induced by cell transfection or intratracheal injection of recombinant adenovirus.

**Results:**

CC16 treatment decreased airway inflammation and histological damage of airway epithelium dose-dependently in HDM-induced asthma model. Airway epithelia apoptosis upon HDM stimulation was noticeably abrogated by CC16 in vivo and in vitro. In addition, upregulation of HMGB1 expression and its related signaling were also detected under HDM conditions, while silencing HMGB1 significantly inhibited the apoptosis of BEAS-2B cells. Furthermore, the activity of HMGB1-mediated signaling was restrained after CC16 treatment whereas HMGB1 overexpression abolished the protective effect of CC16 on HDM-induced airway epithelia apoptosis.

**Conclusions:**

Our data confirm that CC16 attenuates HDM-mediated airway inflammation and damage via suppressing airway epithelial cell apoptosis in a HMGB1-dependent manner, suggesting the role of CC16 as a potential protective option for HDM-induced asthma.

## Background

Asthma is a common chronic respiratory disease affecting over 300 million populations worldwide and has become a major global health challenge (GINA Executive and Science committee 2018). As a complex airway disorder, asthma is characterized by various inflammatory cells-mediated immunity, which promotes excessive inflammation, mucus overproduction, airway hyperresponsiveness, and airway remodeling (Lambrecht and Hamida 2014). Therein, the airway epithelium represents the first-line host barrier against foreign substances, which is closely involved in the initial pathogenesis of asthma (Hammad and Lambrecht (2015); Gon and Hashimoto 2018).

Aeroallergens from house dust mites (HDMs) such as *Dermatophagoides pteronyssinus* are the most prevalent sources of a range of allergens which are highly associated with allergic asthma (Zhang et al. 2018). Inhaled HDMs trigger airway epithelial cells to immediately express pattern recognition receptors (PRRs) especially Toll-like receptor 4 (TLR4) (Hammad et al. 2009; McAlees et al. 2015). Upon allergen recognition, activated and damaged airway epithelia release a variety of proinflammatory cytokines and chemokines, eventually leading to asthma pathogenesis (Lambrecht et al. 2019). In particular, HDM allergen challenge contributes to epithelial cells apoptosis, increased epithelium permeability and histological changes, which finally orchestrates airway injury.

High mobility group box 1 (HMGB1) is an important inflammatory mediator released from injured and death cells and believed to be endogenous danger signal for DNA repair, recombinant, cell death and apoptosis, which is responsible for multiple cancers and immune diseases(Cavone et al. 2015; Kang et al. 2014). It has been reported that HMGB1 critically participates in inflammatory development of asthma by acting as a ligand of TLR4 (Shang et al. 2019). Besides, emerging studies revealed that HMGB1 was elevated in induced sputum and plasma in asthmatic patients, and that measures to inhibit HMGB1 were helpful to alleviate airway inflammation in ovalbumin(OVA)-induced asthma model (Watanabe et al. 2011; Shang et al. 2020). It is likely that HMGB1 plays an important role in allergic pathophysiological process. But the effect of HMGB1 on HDM-induced airway damage and inflammation is not well elucidated.

Clara cell secretory protein(CC16), also known as CC10, uteroglobin, secretoglobin-1A1, or club cell secretory protein(CCSP), is a 16-kDa homodimeric protein belonging to the secretoglobin superfamily and is mainly secreted by mucosal nonciliated airway epithelial (Clara) cells localized in bronchi and bronchioles(Mukherjeea et al. 1999). CC16 possesses anti-inflammatory and immunoregulatory properties, and is regarded as an endogenous protective protein against several pulmonary diseases. Previous studies have shown that recombinant CC16 can help to decrease airway inflammatory response in chronic obstructive pulmonary disease (COPD) and acute respiratory disease syndrome (ARDS) (Pang et al. 2018; Lopez et al. 2020). Given that airway damage and inflammation participate in HDM-induced asthma as well, we proposed that CC16 might serve as a helpful regimen to abrogate injured airway epithelium. Moreover, some studies demonstrated that CC16 could inhibit the transcription factor nuclear factor-κB (NF-κB) signaling pathway in airway inflammatory diseases (Pang et al. 2018; Tokita et al. 2014). It is well known that NF-κB pathway is downstream signaling of HMGB1-TLR4 axis and is able to modulate inflammatory cytokine genes expression in asthma (Poynter et al. 2002). Nevertheless, whether CC16 would protect against proinflammatory effect of HMGB1 remains elusive. In this study, using cultured cells and a HDM-induced murine asthma model, we investigated the participation of HMGB1 together with potential signaling molecules in progression to airway inflammation of asthma. We also explored protective effect of CC16 on airway damage and epithelial cell apoptosis exposed to HDM allergen and the underlying mechanism. This study may shed light on a novel remedial option for HDM-induced asthma.

## Materials and methods

### HDM sensitized and challenged model and recombinant CC16 treatment

Healthy female Balb/c wild-type (WT) mice (6–8 weeks old, 20–25 g) were maintained in pathogen-free animal facility in a 12 h light–dark cycle with regular food and water. In order to explore the preventive effect of CC16 on HDM-induced airway inflammation and injury, the WT mice were randomly divided into four groups and were subjected to the following regimens: (1) Control group; (2) HDM group; (3) HDM + CC16-5 group (treated with CC16 at a dose of 5 μg/g/body); (4) HDM + CC16-10 group (treated with CC16 at a dose of 10 μg/g/body). To establish an animal model of asthma, mice were sensitized with intraperitoneal injection of HDM (*Dermatophagoides pteronyssinus*) extract (Greer Labs, Lenoir, NC, USA) 100 µg in 200 µl phosphate buffered saline(PBS) on Days 0, 7, and 14 respectively. On Day 21–28, mice were challenged by intranasal administration of 100 μg HDM (solved in 50 µl PBS) daily under chloral hydrate anesthesia. Intranasal treatment of CC16 (5 μg/g or 10 μg/g) (PeproTech, Rocky Hill, NJ, USA) (dissolved in 20 µl sterile saline) or the same dose of saline were given 30 min before each HDM challenge. Mice received PBS instead of HDM in the sensitization and challenge phase as negative control. All mice were sacrificed for endpoint analysis on day 35. The bronchoalveolar lavage fluid (BALF) was collected by injecting and retracting 1 ml of 0.9% saline solution and was then centrifuged for cytokines analysis and differential cell counts. The lung tissues were harvested appropriately for subsequent histopathological examination. All murine experimental procedures were approved by the Animal Research Ethical Committee of Shanghai East hospital.

### Adenovirus gene delivery

To determine the in vivo effect of HMGB1 on allergic airway injury, recombinant adenovirus expressing the mouse HMGB1 gene (Ad-HMGB1) or mouse HMGB1 shRNA(Ad-sh-HMGB1) was constructed by OBiO Technology Corp. Ltd. (Shanghai, China). Adenoviral vectors containing no transgene were used as negative control (Ad-GFP). One week before the establishment of asthmatic model, a dose (1 × 10^9^ pfu) of Ad-PGRN, Ad-sh-HMGB1 or Ad-GFP were intratraceally delivered into the mice. The efficacy of interfere was evaluated with qPCR.

### Cell culture

The human airway epithelial BEAS-2B cells were obtained from the cell bank of the Chinese Academy of Science (Shanghai, China) and cultured in DMEM/F medium (Hyclone, Camarillo, CA, USA) containing 10% fetal bovine serum(Clark Bioscience, Claymont, DE,USA), 100 U/ml penicillin and 100 μg/ml streptomycin(Gibco, Grand Island, NY, USA) at 37 °C with 5% CO_2_ in humidified air. After reaching 80–90% confluence, the cells were seeded to proper culture slides and acclimated with free serum free-DMEM for subsequent experimental purpose.

### Cell transfection

In order to knockdown the expression of HMGB1, small-interfering RNAs (siRNAs) targeting human HMGB1 gene (si-HMGB1) and negative control siRNA (si-NC) were synthesized and purchased from GenePharma (Shanghai, China).For overexpressing HMGB1, recombinant pcDNA-HMGB1 plasmid was constructed by cloning the full-length HMGB1 into pcDNA3.1 vectors. Empty vector was used as the negative control. For transfection, BEAS-2B cells were transfected with si-HMGB1, si-NC, pcDNA-HMGB1 or vector using the lipofectamine 2000 according to the manufacturer’s protocol (Invitrogen, Camarillo CA, USA).

### Lung histopathology and TUNEL staining

The lung samples were fixed in 10% formalin overnight and embedded in paraffin. Lung sections of 4 μm thickness were stained with haematoxylin and eosin(H&E), as well as periodic acid Schiff (PAS) for histopathology analysis. TUNEL staining was performed using the InSitu Cell Death Detection kit (Roche, Switzerland, no.11684817910) to detect the apoptotic cells.

### Immunohistochemistry

The tissue samples were dewaxed in xylene and rehydrated in graded ethanol solutions. The sections were blocked with normal goat serum and incubated for 20 min at room temperature. Then the sections were immunostained with the primary antibodies against HMGB1(Cat#6893, Cell Signaling Technology, Danvers, MA,USA) at 4 °C overnight. Followed by washing three times with PBS, the sections were incubated with HRP-labeled secondary antibody at 37 °C for 30 min. The stained sections were observed under a light microscopy.

### Immunofluorescence analysis

The treated BEAS-2B cells were fixed with 4% paraformaldehyde and permeated with 0.1% Triton X-100 for 10 min. After incubation with anti-HMGB1 at 4 °C overnight, the cells were stained with secondary Alexa Fluor®488-conjugated antibody (Beyotime Biotechnology, Shanghai, China) for 1 h at 37 °C in the dark. The nuclei were counterstained with DAPI. Images were visualized by using a fluorescence microscope.

### Cell viability assay

BEAS-2B cells were seeded into a 96-well plate at a density of 3000 cells/well and treated as described above. Then cell viability was assessed with the Cell Counting Kit-8 assay (CCK8; Dojindo Laboratories, Tokyo, Japan) at 0, 12, 24, 48 h, respectively. The optical density(OD) values of the absorbance at 450 nm were measured using Riorad microplate reader.

### ELISA

Measurements of IL-4, IL-5, IL-13 (Abcam, Cambridge, UK), HDM specific-IgE (Chondrex, Redmond, USA) and HMGB1 (Arigobio, Taiwan, China) in the BALFs as well as serum CC16 (Biovendor Systems, Candler, USA) were performed with enzyme-linked immunosorbent assay (ELISA) kits according to the manufacturer’s recommendations.

### Flow cytometry

Cell apoptosis was determined using double staining with Annexin V-FITC/propidium iodide (PI). Briefly, the treated cells from different groups were resuspended in a 500 μl binding buffer volume and 5ul of Annexin V-FITC and 5 μl of PI (Beyotime) were added. Subsequently, the cells were incubated for 15 min at room temperature protected from light. The phycoerythrin (PE)-conjugated anti-cleaved caspase-3(Cat#9661, Cell Signaling Technology) antigen was used to detect activated caspase-3 of cells according to the manufacturer’s protocol. All data were quantified on FACS Calibur flow cytometer (BD Biosciences, CA, USA).

### Western blot analysis

Lung tissues or cells were lysed with RIPA buffer containing protease inhibitor cocktail and phenylmethylsulfonyl fluoride. The supernatants were collected by centrifuged and total protein concentration was qualified using a BCA Protein Kit (Beyotime Biotechnology, Shanghai, China). Then equal quantities of protein samples were separated by 8% SDS-PAGE and transferred onto PVDF membranes (Meckmillipore, Germany) that were consequently blocked with 5% fat-free milk for 1 hr. After that, the membranes were incubated with primary antibodies at 4 °C overnight. The primary antibodies were used as follows: anti-HMGB1 (1:1000; Cat#6893),anti-TLR4 (1:1000; Cat#14358), anti-NF-κB (1:1000; Cat#8242), anti-p-NF-κB (1:1000; Cat#3033), cleaved caspase-3 (1:1000; Cat#9661) (Cell Signaling Technology); anti-Bcl-2 (1:1000; Cat#ab182858), anti-Bax (1:1000; Cat#ab32503), and anti-β-actin (1:1000; Cat#ab8227) (Abcam). Following extensively rinsing in TBST, the membranes were incubated with HRP-conjugated secondary antibodies and further detected using ECL chemiluminescent method (Millipore, Billerica, MA, USA). The blots were quantified with ImageJ.

### RNA extraction and quantitative reverse transcription polymerase chain reaction (qRT-PCR)

Total RNA was extracted from lung tissues or cells with TRIzol reagent (Invitrogen) and diluted in nuclease-free DEPC-treated water. After the preparation with DNase treatment (Qiagen, Hilden, Germany), total RNA was reverse transcribed into cDNA which was applied as a template for qRT-PCR reaction. Then relative mRNA levels were quantified by ABI PowerUp^TM^SYBR™ Green Master Mix (Termo Fisher Scientific, Waltham, MA, USA). PCR conditions consisted of 40 cycles at 95 °C for 15 s and 60 °C for 1 min, followed by a melting curve analysis. All reactions were performed using an ABI7500 Fast Real-Time PCR System (Applied Biosystems, USA). The primer sequences were as followed: interleukins (IL)-25 forward: 5′-CGTCCCACTTTACCACAACC-3′ and reverse: 5′-ACACACACACAAGCCAAGGA-3′; IL-33 forward: 5′-GTACTTTATGCAACTGCGTTCTGG-3′ and reverse: 5′- CAGACATTGCTTTCTGCACTTTTC-3′; thymic stromal lymphopoietin (TSLP) forward: 5′-TTCACTCCCCGACAAAACATTT-3′ and reverse: 5′-TGGAGATTGCATGAAGGAATACC-3′; HMGB1 forward: 5′-GGGTACTGCCTTGCTTGACA-3′ and reverse: 5′- ATCAGACCCTTTCAGGAGGC-3′. The expression of target gene was calculated by the 2^−△△CT^ method and β-actin was used as a housekeeping gene.

### Caspase-3 activity assays

The caspase-3 activity in the lung tissues was evaluated with an assay kit (Beyotime Biotechnology) according to manufacturer’s protocols.

### Statistical analysis

Statistical analysis was performed using the SPSS19.0 software. All data were expressed as mean ± standard error of the mean. The Student’s *t*-test (comparisons between two groups) or one-way ANOVA with Bonferroni post-hoc test were used for analyses. *P* < 0.05 was considered statistically significant.

## Results

### Levels of serum CC16 in mice before and after HDM inhalation

Using ELISA assay, serum CC16 concentrations of the control and HDM groups were detected on Days 0, 7, 21, and 35, respectively. As summarized in Table 1, baseline levels of serum CC16 on Day 0 prior to HDM inhalation did not differ between the two groups. Although the HDM group had slightly higher serum CC16 levels on Day 7 (1 week after initial HDM exposure) than those before HDM sensitization (baseline), the differences were not statistically significant. Compared to baseline, a sharply elevation of serum CC16 levels was observed on Day 21 post-HDM challenge in asthmatic mice. Thereafter, serum levels of CC16 in HDM group on Day 35 were significantly declined to a very low extent, which were even less than the control.

**Table 1:** Serum levels of CC16 in mice before and after HDM inhalation

Serum CC16(ng/ml)	Time course of HDM induction			
	Day 0	Day 7	Day 21	Day 35
Control	2.92 ± 1.77	3.22 ± 1.54	2.79 ± 1.68	3.04 ± 1.66
HDM	2.76 ± 2.15	3.17 ± 1.97	5.36 ± 2.27*	1.31 ± 0.90*

Means ± SEM, n = 6

*p < 0.05 vs. control group

### CC16 alleviates airway inflammation and airway epithelial injury in HDM-induced asthmatic mice

**Fig. 1:**
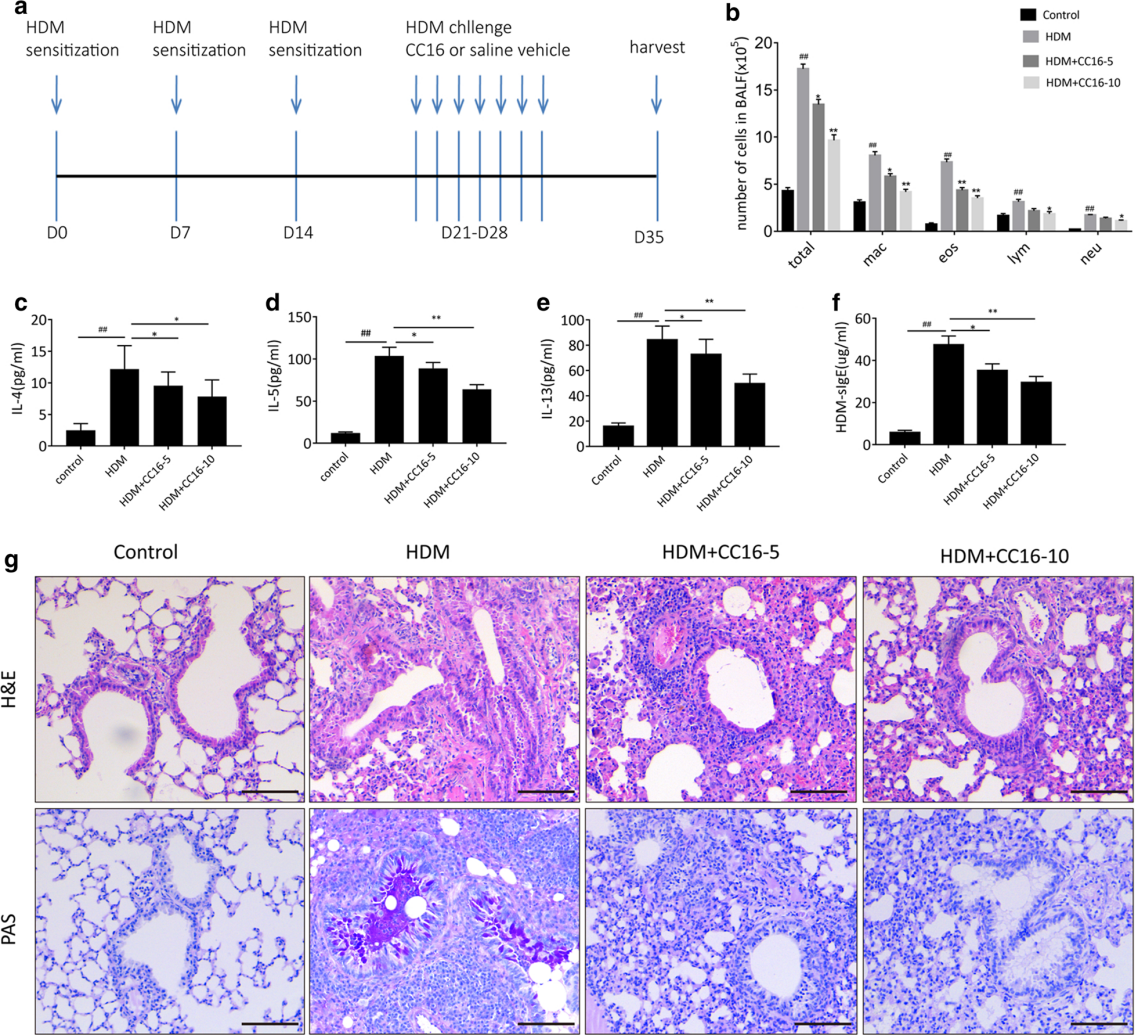
CC16 inhibits HDM-induced airway epithelial injury and inflammation in vivo. **a** Experimental protocol for the establishment of HDM-induced murine asthmatic model and treatment with recombinant CC16. **b** The counts of total and differential inflammatory cells in BALF from control group, HDM group, HDM + CC16-5 (treated with 5 ug/g CC16) group, and HDM + CC16-10 (treated with 10 ug/g CC16) group by Wright staining. **c**–**f** The levels of IL-4, IL-5, IL-13 and HDM-sIgE in BALF were measured using ELISA assay. **g** Representative lung sections from each group were analyzed with H&E or PAS staining. Scale bars = 100 μm. Data are expressed as means ± standard error of the mean (SEM). ^##^p < 0.01 vs. Control group;*p < 0.05 vs. HDM group; **p < 0.01 vs. HDM group

To explore the possible protective role of CC16 in allergic airway inflammation and airway epithelial injury, we established a HDM-induced murine asthmatic model and treated mice with or without ranged doses of recombinant CC16 (5 ug/g/mouse or 10 ug/g/mouse) prior to HDM challenge (Fig. 1a). BALF was collected 24 h after the mice were sacrificed, and then total and differential inflammatory cells were counted. As shown in Fig. 1b, the number of total cells was significantly elevated in HDM-induced asthmatic group compared with that from the saline-challenged group, mainly represented in alveolar macrophages (AM), eosinophils (Eos), lymphocytes (Lym), and neutrophils (Neu). BALF inflammatory cell counts from HDM-challenged mice that received low or high dose of CC16 (5 μg/g, 10 μg/g) were both reduced compared with those from HDM-challenged control. Of note, administration with high dose of CC16 (10 μg/g) exhibited a markedly reduction in the infiltration of BALF inflammatory cells in contrast to low dose of CC16 (10 μg/g). Similarly, compared with the control, the HDM-induced asthmatic group showed a significant elevation in the production of Th2-associated inflammatory cytokines including IL-4, IL-5,IL-13 and HDM-specific IgE (sIgE) via ELISA assay, which were inhibited by CC16 treatment in a dose-dependent manner (Fig. 1c–f).

By histopathological analysis of lung tissues stained with hematoxylin–eosin, our findings showed that HDM challenge resulted in extensive airway wall thickening, mucosal metaplasia as well as the recruitment and infiltration of inflammatory cells in peribronchial and perivascular areas. Further PAS staining exhibited collagen deposition, goblet cell hyperplasia and mucus hypersecretion that constituted airway damage. Intriguingly, asthmatic mice with administration of CC16 had less inflammatory cell infiltration especially eosinophils together with mild histological damage of airway epithelial tissues. Moreover, a greater pathological alleviation in 10 ug/g CC16-treated asthmatic group had been discovered than that in 5 ug/g CC16-treated group (Fig. 1g). All these results indicated that CC16 treatment dose-dependently suppressed HDM-induced airway injury and inflammation.

### CC16 treatment prevents airway epithelia against apoptosis exposed to HDM in mice

**Fig. 2:**
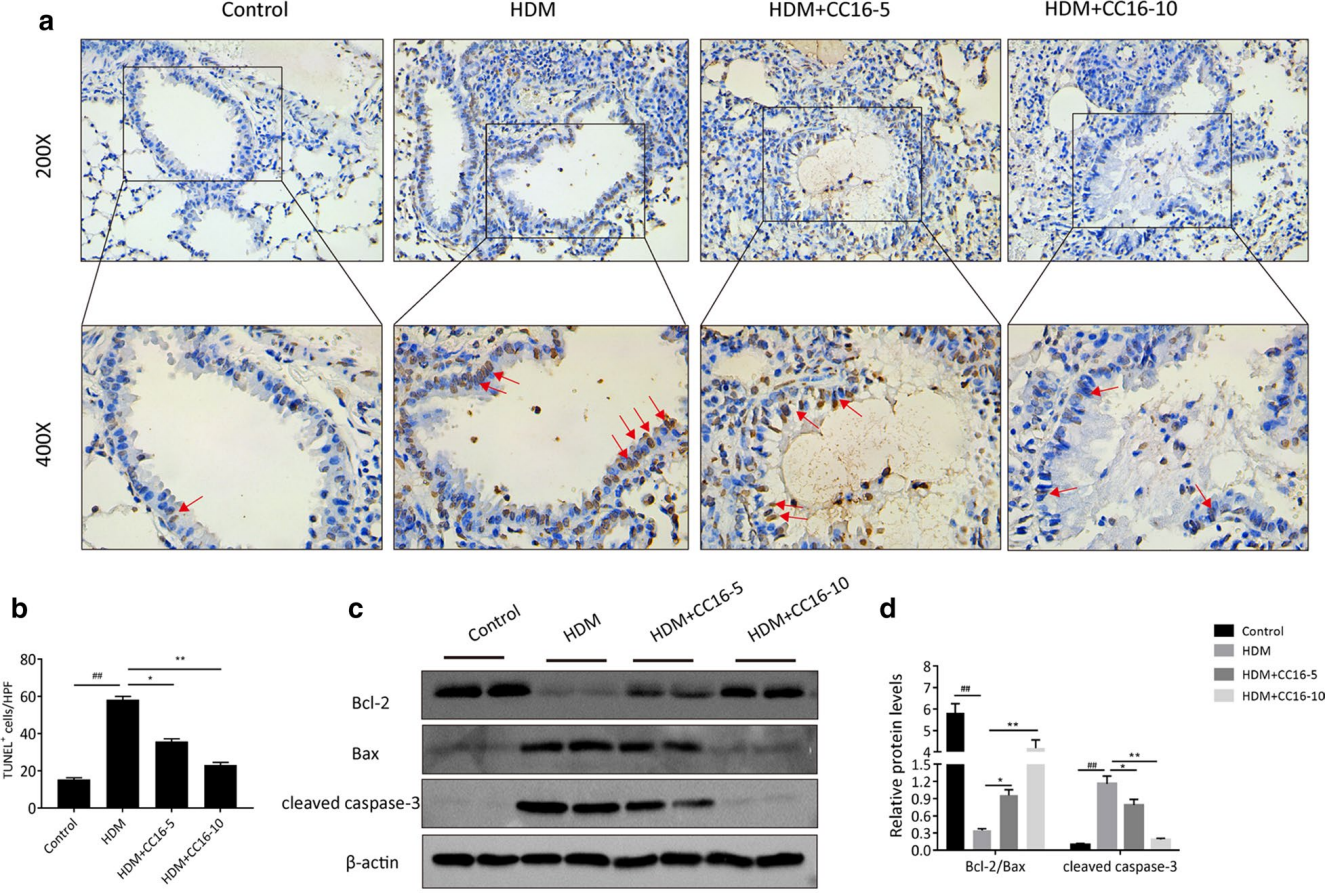
CC16 suppresses HDM-induced airway epithelial apoptosis in mice. **a** Representative staining for TUNEL images from airway epithelial tissues of each group (magnification at × 200 and × 400). **b** Quantitative assessment of the percentage of TUNEL-positive cells in 5 random areas for each section. Red arrows indicated apoptotic airway epithelial cells. **c** Representative immunoblotting images of Bcl-2, Bax and cleaved caspase-3 expressions in lung tissues from each group. **d** Relative expression levels of above proteins was shown as bar graphs. Data are expressed as means ± SEM. ^##^p < 0.01 vs. Control group; *p < 0.05 vs. HDM group; **p < 0.01 vs. HDM group

HDM allergen can induce airway epithelium dysfunction and promote epithelial cells apoptosis that have been considered to be strongly associated with airway injury (Gandhi et al. 2013). To further explore the antiapoptotic impact of CC16 on airway epithelium, we performed TUNEL experiment to evaluate the level of apoptosis in airway tissues under HDM exposure. The quantitation of TUNEL-positive cells was based on the average from five randomly chosen areas per sample. In normal lung, few airway epithelia expressed TUNEL-positive cells. In contrast, HDM challenge significantly augmented the number of TUNEL-positive cells in airway epithelium in asthmatic mice (Fig. 2a), suggesting that HDM allergen caused severe airway damage. Upon pretreatment with CC16 during HDM challenge, especially high-dose CC16 treatment, the ratio of TUNEL-labeled airway epithelial cells was significantly decreased compared with that in HDM alone group (Fig. 2b). Importantly, the extent of TUNEL positivity was parallel to pathological changes of airway epithelium. Thus, epithelial cells apoptosis reflected the degree of airway injury and inflammation.

In order to determine the antiapoptotic regulatory mechanism of CC16, the protein levels of Bcl-2, Bax and cleaved caspase-3 of airway tissues were evaluated by Western blot among different groups (Fig. 2c). Compared to control group, the expression of antiapoptotic protein Bcl-2 was significantly decreased in asthmatic mice under HDM conditions companied with the upregulation of proapoptotic protein Bax and cleaved caspase-3 expression, indicative of HDM allergen-induced propensity to epithelial apoptosis. However, treatment with CC16 exhibited a dose-dependent improvement of Bcl-2/Bax protein ratio and downregulated cleaved caspase-3 expression in contrast to HDM alone (Fig. 2d). These data displayed that CC16 administration might protect against airway epithelial cell injury and apoptosis through inhibiting the activation of mitochondrial apoptotic pathway.

### The cytoprotective effect of CC16 on HDM-induced injury and inflammatory cytokines production in BEAS-2B cells

**Fig. 3:**
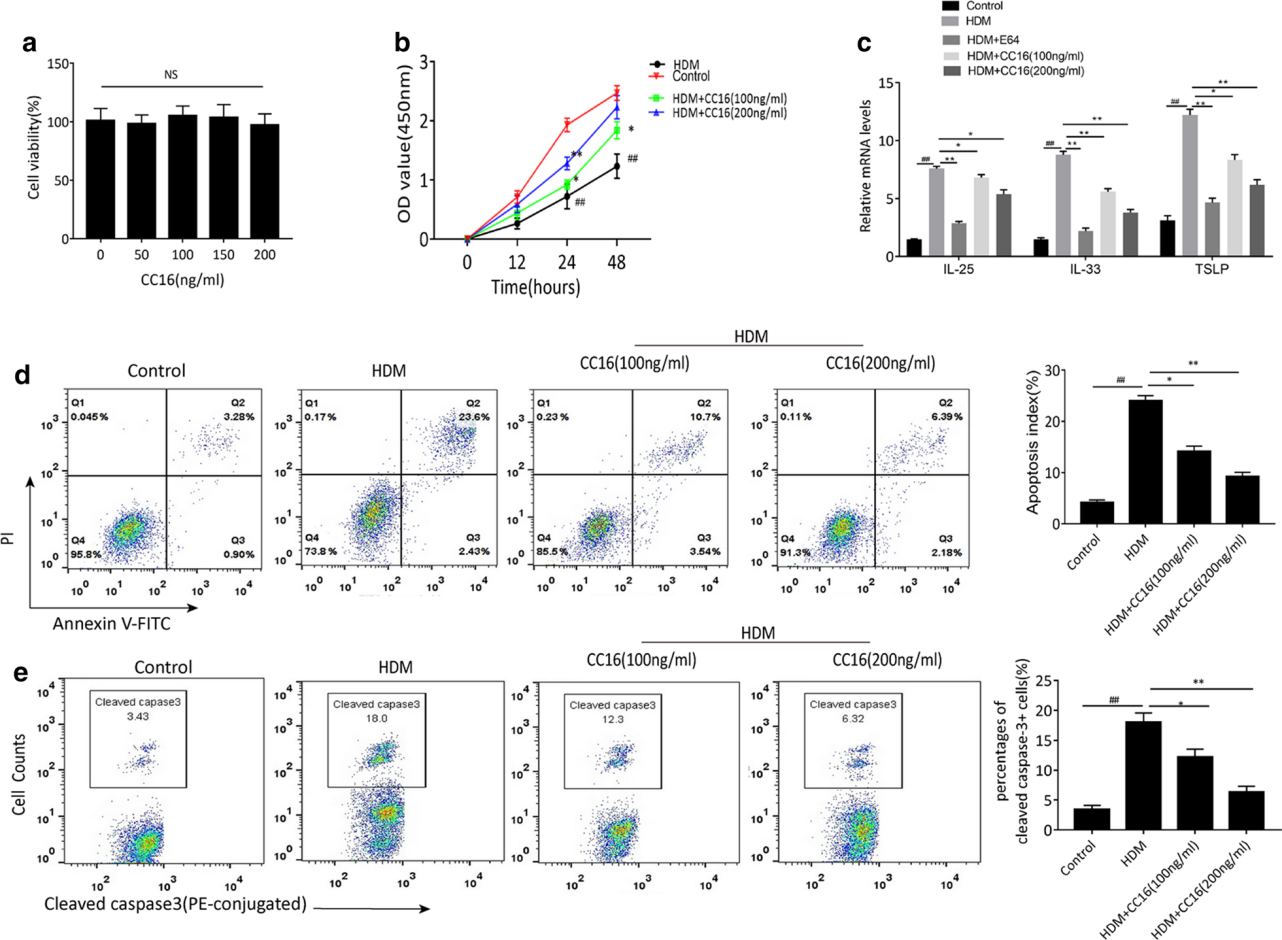
CC16 improves cell viability and reduces apoptosis and inflammatory gene expression in HDM-induced BEAS-2B cells. **a** BEAS-2B cells were incubated in various concentrations of CC16 for 24 h. Cell viability was then analyzed by CCK-8 assay. **b** BEAS-2B cells were pretreated with or without different concentrations of CC16 (100 ng/ml or 200 ng/ml) for 24 h, and then stimulated with 300u/ml HDM. CCK-8 assay was performed to analyze cell viability after HDM stimulation for 12, 24, 48 h (hr). **c** The mRNA expression of IL-25, IL23 and TSLP were detected by RT-qPCR. **d** Flow analysis of apoptotic cells in the control, HDM, HDM + CC16(100 ng/ml), and HDM + CC16(200 ng/ml) groups. **e** Percentages of activated caspase-3 positive cells by flow cytometry in different treatment groups. Data are expressed as means ± SEM. ^##^p < 0.01 vs. Control group; *p < 0.05 vs. HDM group; **p < 0.01 vs. HDM group

HDM-induced airway injury and inflammatory response are closely related to airway epithelial cell dysfunction, which aggravate the pathogenesis of asthma (Shim et al. 2012). To elucidate the protective effect of CC16 in airway epithelia under HDM conditions, we performed experiments on HDM-stimulated normal human airway epithelial BEAS-2B cells. CC16 was applied to pretreat BEAS-2B cells with various concentrations ranged from 5 to 200 ng/ml for 24 h. As a result, CC16 showed little cytotoxicity in BEAS-2B cells at concentrations less than 200 ng/ml (Fig. 3a). Subsequently, BEAS-2B cells were incubated with CC16 (100 ng/ml and 200 ng/ml, respectively) for 24 h and then exposed to 300 ng/ml HDM for 48 h. PBS was used as a negative control. The proliferation of HDM-stimulated BEAS-2B cells was further assessed by CCK-8 assay. As displayed in Fig. 3b, HDM exposure significantly restrained cell viability in comparison with the untreated control cells, while the growth rate of BEAS-2B cells was significantly attenuated by CC16 treatment dose-dependently. Since HDM stimulation contributes to the production of epithelial-derived proinflammatory mediators via its protease activity, BEAS-2B cells were additionally coincubated with HDM (300 ng/ml) and cysteine protease activity inhibitor E64 (20 μM) (Sigma-Aldrich, Stockholm, Sweden). Our results showed increased mRNA levels of epithelial-derived cytokines IL-25, IL-33 and TSLP in HDM-treated BEAS-2B cells compared to control group, whereas they were significantly blocked in the E64-treated group. Noticeably, dose-dependently decreases of IL-25, IL-33 and TSLP expression were also observed in CC16 groups compared to HDM alone group, although the degree of suppression was less than that of E64-treated cells (Fig. 3c).

We also investigated whether CC16 treatment could ameliorate HDM-evoked apoptosis in BEAS-2B cells. The flow cytometry results showed a dramatic elevation in the proportion of apoptotic cells upon HDM stimulation, while CC16 pretreatment reversed HDM-induced BEAS-2B cells apoptosis by 26% and 50%, respectively, according to different doses of CC16 (Fig. 3d). The percentage of cleaved caspase-3 positive cells was also significantly increased in HDM group compared to the control, and declined to varying degrees with different doses of CC16 treatment (Fig. 3e). These data indicate that CC16 exerts an anti-inflammatory and antiapoptotic influence on HDM-induced BEAS-2B cells which is similar to airway tissues described above.

### CC16 suppresses HDM-induced overexpression of HMGB1 in vivo and in vitro

**Fig. 4:**
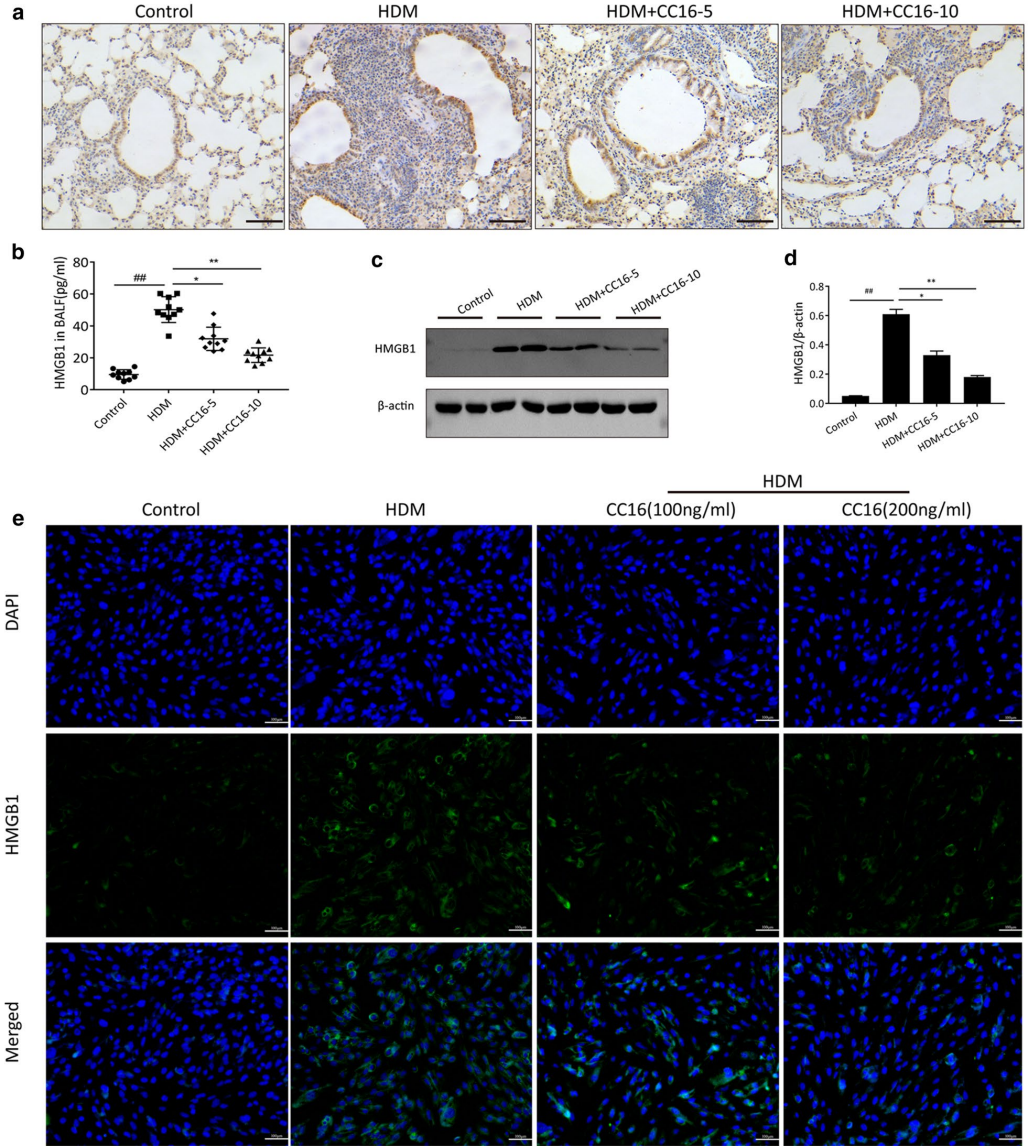
CC16 treatment restrains HDM-induced upregulation of HMGB1 in vivo and in vitro. **a** Representative immunohistochemistry images of HMGB1 expression in airway epithelium after different treatments. Scale bar = 100 μm. **b** The BALF levels of HMGB1 were determined by ELISA among different groups. **c** Western blot was performed to examine HMGB1 expression in lung tissues of different groups. **d** Quantitation of the HMGB1/β-actin ratios. **e** Representative immunofluorescence images of HMGB1 (green) in BEAS-2B cells with different treatments. Scale bar = 100 μm. Data are expressed as means ± SEM. ^##^p < 0.01 vs. Control group; *p < 0.05 vs. HDM group; **p < 0.01 vs. HDM group

Allergens such as HDM can lead to the upregulation of HMGB1 protein by injuried airway epithelial cells (Lambrecht and Hammad 2014). Many studies have confirmed that HMGB1 plays an essential role in allergic airway inflammation as a signal for DNA repair and cell death. In view of CC16-mediated protective effect on HDM-challenged airway epithelial cells damage, we hypothesized that regulation of HMGB1 expression might be involved in the underlying molecular mechanism of CC16 treatment. In the current study, the expression of HMGB1 was investigated in vitro and in vivo. In the HDM-induced asthma model, immunochemistry findings showed that HMGB1 was mainly expressed in airway epithelium and some peripherally infiltrative lymphocytes of lung tissues, suggesting that airway epithelial cell was an important source of HMGB1 production. In particular, HMGB1 expression was distinctly detected in the nuclei and cytoplasm of airway epithelia in HDM-challenged asthmatic group, whereas HMGB1 was only weekly or modestly stained in airway epithelia nuclei in control group (Fig. 4a). These results illustrated that HDM could induce HMGB1 to be translocated from the nucleus to the cytoplasm. Furthermore, the elevated expression especially cytoplasmic HMGB1 in HDM-induced mice was partially diminished by CC16 administration, with better improvement observed in high-dose CC16-treated group. In addition, HMGB1 protein levels in BALF and lung tissue were evaluated by ELISA assay and Western blot respectively. As shown in Fig. 4b–d, HMGB1 expression was significantly increased in both the BALF and lungs of asthmatic mice in contrast to those in control group, indicating that extracellular release of HMGB1 was also actively promoted after HDM exposure except for nucleocytoplasmic translocation. As expected, these changes of HMGB1 expression were reversed by pretreatment with CC16 in a dose-dependent manner. To focus on the modulatory function of CC16 on HMGB1 expression in airway epithelial cells, immunofluorescence assay was conducted to detect the cellular localization of HMGB1 protein following the in vitro HDM-mediated damage. Likewise, HDM-challenged BEAS-2B cells showed significantly increased cytoplasmic and extracellular expression of HMGB1 whilst a little faint HMGB1 immunofluorescence staining was detected in the nuclei of PBS-treated control cells. In contrast, HDM + CC16 group showed a markedly reduction of HMGB1 staining as compared with HDM group, suggesting that HDM-stimulated HMGB1 upregulation was dramatically abolished by CC16 especially extracellular HMGB1 release, as displayed in Fig. 4e. Moreover, the cells treated with high-dose CC16 showed less HMGB1 expression than CC16 low-dose group. Altogether, based on these data, it was referred that CC6 could suppress HDM-induced HMGB1 activation in airway epithelial cells.

### HMGB1 contributes to HDM-induced airway epithelia damage through TLR4/NF-κB signaling pathway

**Fig. 5:**
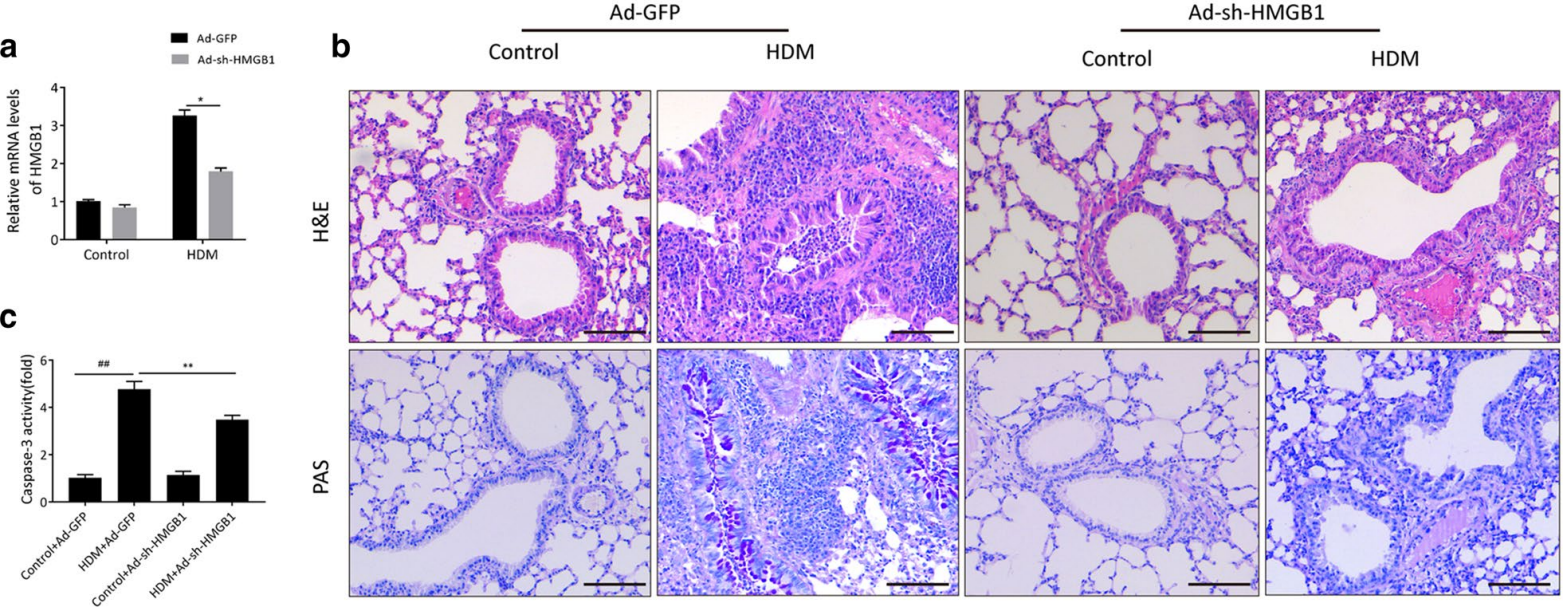
HMGB1 deficiency alleviates airway damage in HDM-induced mice. Ad-GFP and Ad-sh-HMGB1 mice were generated by intratracheally delivery of recombinant adenovirus, and then subjected to saline (negative control) or HDM exposure. **a** Relative mRNA levels of HMGB1 in mouse lungs. **b** Histopathological analysis of lung tissue sections by H&E or PAS staining. **c** The caspase-3 activities of the lungs in different treatment groups. Data are expressed as ^##^p < 0.05 vs. control + Ad-GFP group; *p < 0.05 vs. HDM + Ad-GFP group; **p < 0.01 vs. HDM + Ad-GFP group

It is well accepted that TLR4, a crucial PRR interacted with HMGB1, is generally expressed by airway epithelial cells in response to inhaled HDM allergen and is required for the subsequent activation of NF-κB that modulates apoptosis and inflammatory cytokine genes. Since HDM allergen led to upregulation of HMGB1 expression and the latter was negatively regulated by CC16, we next explored the role of HMGB1 as well as its potential signal pathway involved in HDM-mediated airway epithelial cell injury and apoptosis. To determine the in vivo effect of HMGB1 on HDM-induced allergic airway injury, HMGB1 expression was intervened via intratracheally administration of Ad-sh-HMGB1 or Ad-GEP vector before the establishment of asthma model. As shown in Fig. 5a, relative mRNA level of HMGB1 was markedly downregulated in airway tissues of the mice receiving Ad-sh-HMGB1 relative to the Ad-GFP-treated mice under both PBS and HDM conditions. Histological analysis revealed that there was a noticeable augmentation in inflammatory cells infiltration and airway wall thicken in Ad-GEP-treated mice with HDM inhalation compared to those receiving Ad-GEP alone. However, in contrast with that in the HDM + Ad-GEP group, administration of Ad-sh-HMGB1 into the lungs of mice exhibited relatively less severe damage of airway tissues under HDM conditions (Fig. 5b). Similarly, HDM remarkably promoted caspase-3 activity in the lungs of Ad-GEP-treated group, which was partially decreased by shRNA-mediated HMGB1 knockdown (Fig. 5c).

**Fig. 6:**
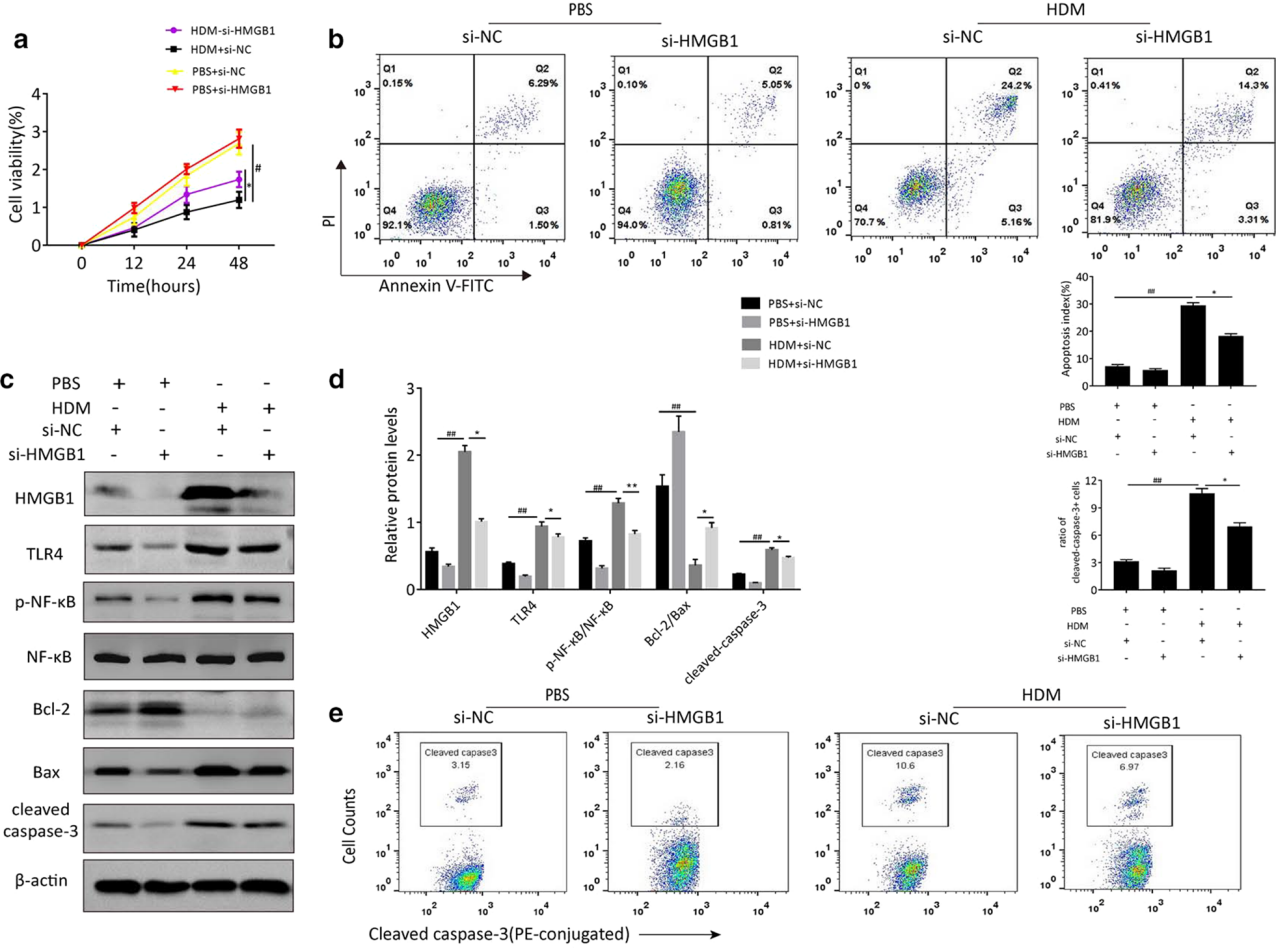
HMGB1 drives airway epithelial cell apoptosis through TLR4/NF-κB signal pathway in HDM conditions. BEAS-2B cells were transfected with si-NC or si-HMGB1, and then incubated in the presence or absence of HDM stimulation. **a** Cell viability of si-NC or si-HMGB1 transfected BEAS-2B cells was assessed by CCK-8 assay after HDM stimulation for 12, 24, 48 h respectively. **b** The percentages of apoptosis cells were measured by flow cytometry. **c** Expressions of HMGB1, TLR4, NF-κB, p-NF-κB, Bcl-2, Bax and cleaved caspase-3 were analyzed by Western Blotting analysis. The level of β-actin served as a loading control. **d** Grey values of the indicated proteins were quantified by densitometric analysis. **e** The percentages of cleaved caspase-3 positive cells by flow cytometry. Data are shown as mean ± SEM. ^#^p < 0.05 vs. PBS + si-NC group (control group); ^##^p < 0.01 vs. PBS + si-NC group (control group); *p < 0.05 vs. HDM + si-NC group; **p < 0.01 vs. HDM + si-HMGB1 group

In the in vitro experiment, following transfected with si-NC and si-HMGB1 respectively, BEAS-2B cells were incubated in the presence or absence of HDM stimulation for 12 h. As expected, HDM stimulation indeed suppressed the growth rate of BEAS-2B cells in contrast to that of PBS-treated control cells via CCK-8 assay. Simultaneously, transfection with si-HMGB1 effectively attenuated the decrease of cell viability caused by HDM (Fig. 6a). Accordingly, for flow-cytometry assay, it was found that silencing HMGB1 remarkably antagonized the facilitative effect of HDM on cell apoptosis (Fig. 6b). There was no significant difference between PBS + si-NC group and PBS + si-HMGB1 group. These findings suggested that HMGB1 was essential during the process of HDM-induced cell apoptosis.

Western blot analysis was performed to determine the alterations of the signaling proteins including TLR4, NF-κB and phosphorylated(p)-NF-κB under the mimic asthmatic condition. As shown in Fig. 6c and d, HDM exposure significantly led to overexpression of HMGB1 that was associated with the upregulation of TLR4/p-NF-κB axis and corresponding changes of apoptosis-related markers compared with control cells. At the same time, si-HMGB1 transfection obviously blocked the increased expression of HMGB1 in BEAS-2B cells induced by HDM, concomitant with the downregulation of TLR4 and p-NF-κB expression. The difference between PBS + si-NC and PBS + si-HMGB1 groups was slight significant. Besides, compared with PBS + si-NC group, a higher level of caspase-3 cleavage was observed by flow cytometry in HDM-treated cells with si-NC transfection, yet decreased by si-HMGB1 transfection (Fig. 6e). Overall, these data demonstrated that HMGB1-mediated airway epithelial cell apoptosis was correlated with TLR4/NF-κB signaling activation in the in vitro model of HDM-induced asthma.

### HMGB1 signaling is involved in CC16-mediated cytoprotection in airway epithelial cells exposed to HDM

**Fig. 7:**
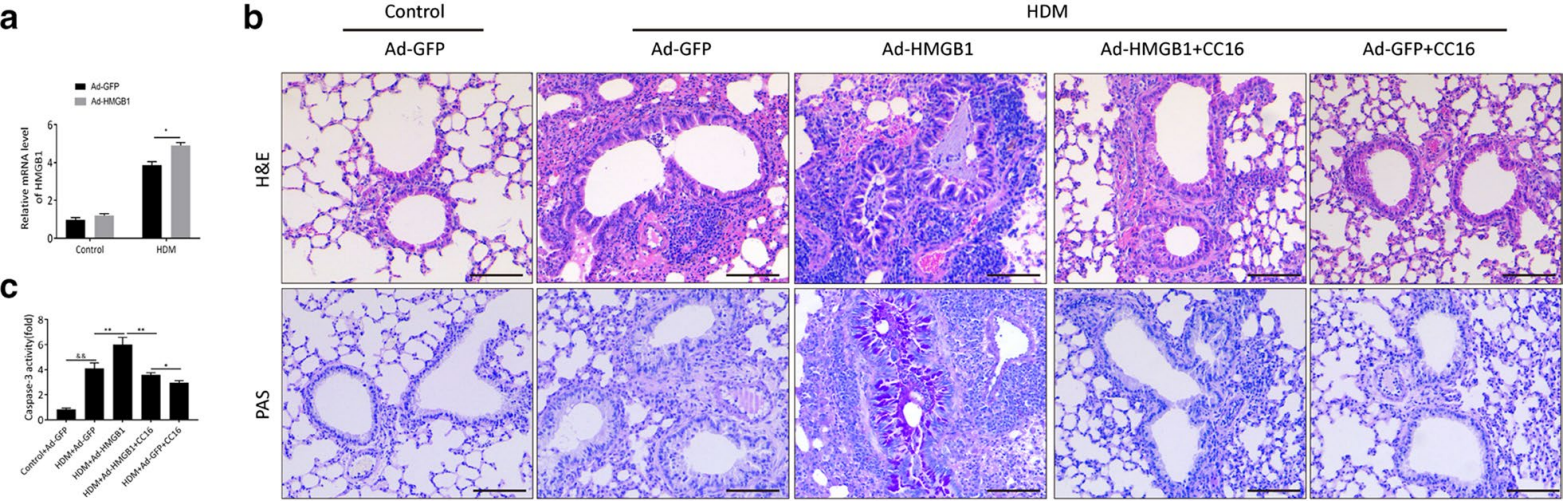
HMGB1 overexpression abolishes the protective effect of CC16 against HDM-induce airway damage. HMGB1 overexpression in mouse lungs was induced by intratracheally injection of Ad-HMGB1 vector. Ad-GFP was used as the negative control as shown in Fig. 5. Then the gene-modified mice was sensitized and challenged by HDM with or without CC16 (10 g/g) administration. **a** Relative mRNA levels of HMGB1 in mouse lungs from different groups. **b** Representative images of lung tissues showed airway wall thickening, mucous hyperplasia and collagen deposition. **c** The caspase-3 activities of the lungs in different treatment groups. Data are expressed as ^&&^p < 0.01 vs. control + Ad-GFP group; ^#^p < 0.05 vs. HDM + Ad-GFP group; ^##^p < 0.01 vs. HDM + Ad-GFP group; *p < 0.05 vs. HDM + Ad-HMGB1 + CC16 group; **p < 0.01 vs. HDM + Ad-HMGB1 + CC16 group

**Fig. 8:**
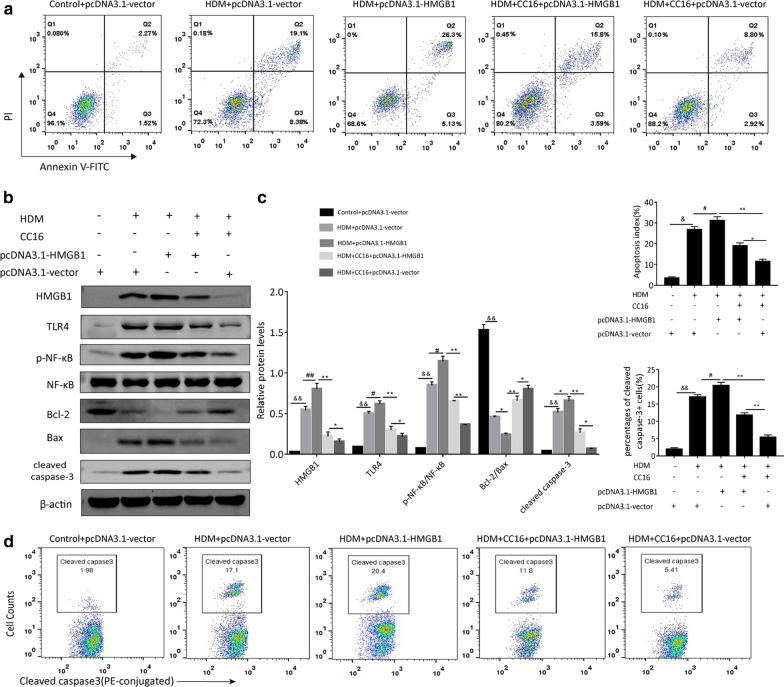
CC16 modulates airway epithelial cell apoptosis in response to HDM through the inhibition of HMGB1-mediated signaling. BEAS-2B cells were transfected with pcDNA3.1-HMGB1 plasmid or pcDNA3.1-vector, and then treated with HDM or CC16 or HDM + CC16 for 48 h respectively. **a** Flow cytometry analysis of HDM-induced apoptosis in BEAS-2B cells. **b** Western Blotting analysis of HMGB1, TLR4, NF-κB, p-NF-κB, Bcl-2, Bax in BEAS-2B cells under different experiments. **c** Quantification of the indicated proteins normalized to GAPDH were shown as bar graphs. **d** Flow cytometry detection of caspase-3 activation in BEAS-2B cells. &p < 0.05 vs. PBS + pcDNA3.1-vector group (control group); ^&&^p < 0.01 vs. control group; ^#^p < 0.05 vs. HDM + pcDNA3.1-vector group; ^##^p < 0.01 vs. HDM + pcDNA3.1-vector group; *p < 0.05 vs. HDM + CC16 + pcDNA3.1-vector group; **p < 0.01 vs HDM + CC16 + pc DNA3.1-vector group

As mentioned above, CC16 could negatively regulate the expression of HMGB1 which imperatively contributed to HDM-mediated airway epithelia damage. To further ascertain whether CC16 exerted protective influence in a HMGB1-dependent manner, the HMGB1-overexpressing mice were generated by the intratracheal injection of Ad-HMGB1 vector prior to HDM exposure. As shown in Fig. 7a, Ad-HMGB1-injected mice had significantly higher mRNA HMGB1 levels in the lungs than GFP-injected mice. Then the HMGB1-modified mice were treated with CC16 (10 g/g) administration or the negative vehicle in response to HDM. Our results demonstrated that CC16 treatment could effectively alleviate airway epithelium edema and mucus production in the mice receiving Ad-GFP under HDM conditions, whereas HDM-induced inflammatory responses and airway damage did not have a great improvement in Ad-HMGB1-injected mice after CC16 administration (Fig. 7b). Caspase-3 activity assay also confirmed that HMGB1 overexpression abated the protective effect of CC16 on HDM-induced apoptosis in mice (Fig. 7c). Additionally, BEAS-2B cells were transfected with the recombinant pcDNA3.1-HMGB1 plasmid to elevate HMGB1 expression. The mechanisms underlying the association between HMGB1-mediated signaling and CC16 were then excavated by Western blot. Consistently, flow-cytometry analysis showed that the increased apoptosis index of BEAS-2B cells exposed to HDM (300 ng/ml) was abrogated by 200 ng/ml CC16 treatment. HMGB1 overexpression abolished the antiapoptotic effect of CC16 on HDM-induced BEAS-2B cells, as proved by the enhancement of cellular apoptosis index (Fig. 8a). The immunoblotting results further displayed that HDM stimulation significantly enhanced protein expression of HMGB1, TLR4 as well as p-NF-kB compared with the pcDNA3.1-vector control group. In contrast to the HDM + pcDNA3.1-vector group, overexpression of HMGB1 rendered an enhancement of HMGB1-mediated TLR4/NF-κB signaling activity caused by HDM. Meanwhile, CC16 treatment markedly reversed the increased activation of HMGB1 signaling in the cells exposed to HDM, suggesting that CC16 acted as a key agent of HMGB1-mediated signaling molecules leading to HDM-evoked inflammation and damage. More importantly, it was found that rescued HMGB1 level by transfection with recombinant plasmid blunted CC16-mediated inhibition on HMGB1/TLR4/NF-κB signaling. The downstream apoptosis regulators of HMGB1-TLR4/NF-κB axis were also detected by immunoblotting analysis. As expected, the ratio of Bcl-2/Bax in HDM-induced group was dramatically lower than that in the control, while cleaved caspase-3 expression level was higher. Accordingly, the alterations in the expression of Bcl-2, Bax, and cleaved caspase-3 upon HDM exposure were abolished by CC16 treatment. However, the antiapoptotic ability of CC16 was subsequently compromised by HMGB1 overexpression, in keeping with the augmentation of HMGB1-mediated TLR4/NF-κB signaling activity (Fig. 8b and c). Flow cytometry of cleaved caspase-3 detection also demonstrated that the adverse effect of CC16 on HDM-induced caspase-3 activation was overturned after the overexpression of HMGB1 (Fig. 8d).

Collectively, our findings indicate that CC16 alleviates HDM-activated airway epithelial injury and apoptosis via inhibition of HMGB1 expression, while HMGB1-mediated signaling proteins such as TLR4 and NF-κB are potentially modulators involved in the airway protection of CC16.

## Discussion

In this study, we demonstrated for the first time that CC16 attenuated the HDM-induced airway inflammatory damage via suppressing airway epithelial cell apoptosis. Most importantly, we have detected that HMGB1 expression and subsequent TLR4/NF-κB signaling activation upon HDM exposure in airway epithelial cells was repressed by CC16 treatment, thus contributing to its anti-inflammatory and antiapoptotic protection. These findings provided insight into the functional role of CC16 as a novel protective agent to control HDM-induced asthma.

CC16 is a small anti-inflammatory protein mainly expressed by nonciliated Clara cells in airway epithelium (Hellings and Steelant 2020).The expression of CC16 is regulated by various factors. Glucocortocoids (Mukherjee et al. 2007) and proinflammatory cytokines such as INF-γ (Magdaleno et al. 1997) and TNF-α (Yao et al. 1998) promote CC16 expression in the airways, whilst exposing to air toxicants such as cigarette smoking(CS) (Robin et al. 2002), nitrogen dioxide (Barth and Müller 1999),and ozone (Royce and Plopper 1997) reduce the synthesis of CC16 by Clara cells. Additionally, CC16 is regarded as a useful biomarker of airway epithelial damage in chronic upper and lower respiratory diseases (Irander et al. 2012; Špadijer-Mirković et al. 2016). Accumulating evidence have confirmed that the number of Clara cell protein-positive epithelial cells is diminished in allergic airway diseases such as asthma, allergic rhinitis and chronic rhinosinusitis (Peric et al. 2018), leading to lower level of CC16 production in BALF and airway mucosa. Another study documented that OVA-challenged CC16^−/−^ mice exhibited more severe airway dysfunction than wild mice (Wang et al. 2001). Also, a low concentration of CC16 in gastric fluid at birth was thought to be correlated with increased lung inflammation in the neonatal period (Hagman et al. 2018).Thus, on the basis of detailed prior studies about the role of CC16 in airway diseases, we postulated that supplementary CC16 might restore airway inflammation in HDM-induced asthma. In the current study, by the establishment of an experimental model of murine asthma, we firstly measured serum CC16 levels in both control and HDM groups. Baseline levels of serum CC16 did not differ between the two groups prior to HDM sensitization. It was of interest to note that serum CC16 levels of asthmatic mice were significantly increased upon the first HDM challenge compared to baseline, yet maximally declined to a very low extent 1 week after the last HDM challenge. These findings were similar to those of a recent study that serum CC16 concentrations were rapidly elevated in asthmatic subjects at 1 h post-allergen challenge (Stenberg et al. 2018). It can be assumed that transient augmentation of serum CC16 following acute HDM challenge is mainly due to allergen-induced increased permeability and diffusion from the epithelial lining fluid into the circulation. But repeated HDM irritations eventually result in the loss of CC16 in the airways. In addition, our findings demonstrated that exogenous administration of CC16 decreased inflammatory cells counts and Th2 cytokines levels including IL-4, IL-5, IL-13 as well as HDM-specific IgE in the BALF of asthmatic mice. Thereby, in line with previous observations (Hung et al. 2004), our study supported that CC16 reduced allergic airway inflammation by inhibiting Th2-type immune response.

The interaction of HDM allergen inhalation and airway epithelial cells has been shown to directly cause airway epithelium dysfunction (Heijink et al. 2020). Due to the activation of airway epithelial cells responding to HDM allergen, a series of epithelial-derived cytokines are rapidly released, and simultaneously recruit neighboring innate and adaptive immune cells, which are essential for the pathogenesis of asthma. Amongst these cytokines, IL-25, IL-33 and TSLP are described as early epithelial-derived alarmins (Mitchell and O’Byrne 2017), for they are markedly released following airway epithelia activation and damage which is a cue for airway epithelial cell death. Moreover, the release of IL-25, IL-33 and TSLP can initiate innate immunity via activating type 2 innate lymphoid cells (ILC2s) (Hammad and Lambrecht 2015). In this study, we found restored levels of IL-25, IL-33 and TSLP in HDM-induced airway epithelial cells followed by CC16 treatment, in line with the alterations in the presence of HDM allergen-related cysteine protease activity inhibitor. Insufficient clearance of impaired apoptotic epithelial cells has previously been documented to lead to the overproduction of IL-33 (Penberthy et al. 2014), which is a crucial substrate for caspases (caspase-3 and -7) activated during apoptosis (Luthi et al. 2009).Another study showed that IL-25 expression was positively associated with the number of apoptotic cells in HDM-induced asthma (Yuan et al. 2017). Increased levels of IL-33, TSLP, and IL-25 in BALF or lung homogenates were also found in OVA-induced asthmatic mice, importantly accompanied with apoptosis of airway epithelium (Sozmen et al. 2016; Lv et al. 2018). Although the detailed relationship between these cytokines and apoptosis was not explicated in the current study, the reduction of the epithelial-derived alarming cytokines implied that CC16 might block airway epithelial activation and injury in respond to HDM, indirectly reflecting the alleviation of innate immune response and epithelial cell death in asthma. Furthermore, histopathological evaluation of airway tissues in our study determined that CC16 administration protected against HDM-triggered damage in airway epithelium, as shown by an observed improvement in airway wall thickening, mucosal metaplasia and mucus hypersecretion. Therefore, we proposed that CC16 might have a repair ability to suppress airway epithelium damage under HDM insult.

Of note, airway apoptosis is implicated in asthma-associated pathophysiology and has been considered to be a vital cause contributing to airway injury and deterioration (Sauler et al. 2019; Lambrecht and Hammad 2013). A report from Tze Khee Chan et al. indicated that HDM-induced allergic asthma resulted in a significant enhancement in DNA damage and apoptosis in lung tissues, especially in airway epithelium (Chan et al. 2016). Besides, apoptotic airway epithelial cells clusters have also been observed in BALF and sputum of asthmatic subjects in contrast to healthy controls (Penberthy et al. 2014).For that reason, we speculated that CC16 might facilitate the repair of airway epithelium damage through the inhibition of airway epithelial apoptosis. Our findings in TUNEL staining displayed that CC16 treatment efficiently abrogated cellular apoptosis in airway epithelium under HDM-challenged conditions. Correspondingly, CC16 treatment also led to a reduction in apoptosis index of BEAS-2B cells exposed to HDM in vitro. Thereof the antiapoptotic property of CC16 in HDM-induced asthma is well documented. In terms of cell death, a recent study showed that brain injury-associated cortical pyroptosis, another form of cell death, was suppressed by CC16 treatment as well (Zhou et al. 2019). It indicates that CC16 may affect more than one mode of cell death except for apoptosis. But whether CC16 regulates pyroptosis in asthma and its underlying mechanism remains to be explicated with further experiments.

As mentioned, upon the stimulation of inhaled allergens such as HDM, activated airway epithelial cells can express PRRs to respond to damage-associated molecular patterns(DAMPs) released upon tissue damage and cell death, accompanied with an increase of endogenous danger signals such as HMGB1 (Mandke and Vasquez 2019). Actually, HMGB1 is identified as a crucial mediator which drives inflammatory response via interacting with TLRs and the receptor for advanced glycation endproducts (RAGE) in several immunological diseases (Liang et al. 2015a; Liu et al. 2020). For another, TLR4 is an acknowledged receptor generally expressed on airway epithelial cells triggered by endogenous danger signals in asthma pathophysiology. The interaction of HMGB1 and TLR4 exposed to HDM allergen contributes to proinflammatory cytokines release through NF-κB signaling transduction (Tracey 2005). In asthmatic patients, HMGB1 sputum levels were positively accordance with the severity of disease. Pharmacological neutralizing HMGB1 was found to suppress asthma-related inflammation and airway remodeling (Lee et al. 2013; Liang et al. 2015b; Hou et al. 2014). Furthermore, HMGB1-mediated signaling pathway is closely relevant to the regulation of tissue injury and cellular apoptosis (Mandke and Vasquez 2019; Gwak et al. 2012). Based on the above ideas, our study was undertaken to assess the expression level of HMGB1 and the link between CC16 and HMGB1 signaling. Consistent with aforementioned researches (Candia et al. 2017), we found an elevated expression of HMGB1 protein in airway epithelium and BALF in HDM-challenged asthmatic mice. The adenovirus-mediated knockdown of HMGB1 effectively attenuated inflammatory infiltration and airway epithelium destruction in mice post-HDM inhalation. By anti-HMGB1 siRNA, the in vitro results showed that HMGB1 downregulation dramatically reduced HDM-induced epithelial cell apoptosis, combined with the inactivation of HMGB1-TLR4/NF-κB signaling. These observations allowed us to raise the possibility that HMGB1 actively takes part in HDM-triggered airway epithelia damage. It was noteworthy that HMGB1 was normally localized in the nuclei of airway epithelial cells, yet translated from the nuclei into the cytoplasm and extracellular space after HDM exposure. Of special interest was that CC16 administration sharply abolished the upregulation of HMGB1 both in nucleocytoplasmic translocation and extracellular release. In addition, HMGB1-mediated TLR4/NF-κB signaling was directly restrained by CC16 as well. Interestingly, although few studies have assessed whether CC16 exerts its effect on cells through binding to a receptor (Laucho-Contreras et al. 2016), a study of lipopolysaccharide-induced acute lung injury indicated that CC16 suppressed TLR4 expression levels on lung macrophages (Snyder et al. 2010), which was similar to our observations. Moreover, CC16 has been identified to as an inhibitor of NF-kB signaling suppress airway inflammation in cigarette smoke-induced chronic obstructive pulmonary diseases from a previous report by Pang et al. (2015). In this study, we detected the propensity of CC16 to modulate HMGB1, which may be a key upstream regulator for NF-κB signaling transduction. Given the importance of extracellular activity of HMGB1 in DAMPs and its nuclear functions as a signal for cell death, we hypothesized that the defensive role of CC16 in airway epithelium damage was highly dependent on the inactivation HMGB1-mediated signaling pathway. To ascertain it, a rescue experiment was conducted to investigate the effect of CC16 in the presence of HMGB1 overexpression. The in vivo study showed that HDM-mediated asthmatic airway inflammation was significantly aggravated in HMGB1-overexpressing mice, although partly improved by CC16 treatment. We also found that antiapoptotic impacts of CC16 on HDM-induced BEAS-2B cells were apparently compromised after transfected with recombinant pcDNA3.1-HMGB1 plasmid. This study determines a relationship between CC16 and HMGB1 signaling in HDM-induced asthma, indicating that CC16 seems to be a potent protector of airway epithelium dysfunction via negatively regulating HMGB1 signaling.

## Conclusion

Taken together, in HDM-induced asthma, HMGB1 signaling pathway is activated, resulting in airway inflammation and apoptosis. CC16 renders a promising anti-inflammatory and antiapoptotic property against airway epithelium damage in a manner dependent on the inhibition of HMGB1 signaling. Overall, our study suggests a potential role of CC16 serving as a novel preventive strategy for HDM-associated allergic airway diseases.

## Data Availability

The data used to support the findings of this study are available from the corresponding author.

## Abbreviations

CC16: Clara cell 16KD protein
HDM: House dust mite
HMGB1: High mobility group box protein 1
BALF: Bronchoalveolar lavage fluid
OVA: Ovalbumin
PRRs: Pattern recognition receptors
TLR4: Toll-like receptor 4
IL: Interleukins
TSLP: Thymic stromal lymphopoietin
ILC2s: Type 2 innate lymphoid cells
DAMPs: Damage-associated molecular patterns
RAGE: Receptor for advanced glycation endproducts
